# Film mulching counteracts the adverse effects of mild moisture deficiency, and improves the quality and yield of *Cyperus esculentus*. L grass and tuber in the oasis area of Tarim Basin

**DOI:** 10.3389/fpls.2024.1296641

**Published:** 2024-04-22

**Authors:** Ya Ding, Zhihao Zhang, Yan Lu, Li Li, Waqar Islam, Fanjiang Zeng

**Affiliations:** ^1^College of Ecology and Environment, Xinjiang University, Urumqi, China; ^2^State Key Laboratory of Desert and Oasis Ecology, Xinjiang Institute of Ecology and Geography, Chinese Academy of Sciences, Urumqi, China; ^3^State Key Laboratory of Desert Plant Roots Ecology and Vegetation Restoration, Xinjiang Institute of Ecology and Geography, Chinese Academy of Sciences, Urumqi, China; ^4^Cele National Station of Observation and Research for Desert-Grassland Ecosystems, Cele, China; ^5^University of Chinese Academy of Sciences, Beijing, China

**Keywords:** arid regions, crude protein, *C. esculentus*, desert ecosystem, nutrients management, ether extract, soluble solids

## Abstract

**Introduction:**

Plastic film mulching (PFM) and deficit irrigation (DI) are vital water-saving approaches in arid agriculture. *Cyperus esculentus* is a significant crop in dry zones. However, scant data exists on the impacts of these water-saving methods on *C. esculentus* yield and quality.

**Method:**

Using randomized block experiment design. Three irrigation strategies were tested: CK (standard irrigation), RW20 (20% water reduction), and RW40 (40% water reduction). Mulchin treatments included film mulching (FM) and no film mulching (NFM).

**Results:**

Results revealed substantial effects of film mulching and drip irrigation on soil nutrients and physical properties, with minor influence on grass, root, and tuber stoichiometry. PF treatment, DI treatments, and their interaction significantly affected *C. esculentus* forage and tuber yields. Initially, grass and tuber yields increased and then decreased with reduced irrigation. The highest yields were under RW20 (3716.31 and 4758.19 kg/ha). FM increased grass and tuber yield by 17.99% and 8.46%, respectively, over NFM. The water reduction augmented the biomass distribuiton of the leaf and root, while reducing the tuber biomass in NFM. FM significantely impacted grass ether extract content, while reduced water influenced grass and tuber crude protein and tuber ether extract content. Mild water stress increased ether extract, crude protein, and soluble matter in grass and tubers, while excessive RW decreased them.

**Conclusion:**

Integrating soil traits, nutrients, yield, and quality, findings indicate *C. esculentus* yield and quality primarily hinge on soil water content, pond hydrogenase, and electrical conductivity. Based on this results, the recommended strategy is to reduce irrigation by 20% for cultivating *C. esculentus* in this area.

## 1 Introduction

Water scarcity, driven by climate change and growing populations, is one of the biggest global challenges (Saha et al., 2016; Pan et al., 2020; Varol, 2020). Notably, climate change intensifies irrigation water deficits in arid and semi-arid regions, amplifying drought occurrence and frequency (Amer, 2010; Chen et al., 2021). Unquestionably, fostering irrigation-efficient agriculture and adopting judicious management strategies to optimize water utilization assume a pivotal role in safeguarding global food security. Water conservation practices, such as plastic film mulching (PFM) and drip irrigation (DI), are widely adopted in agriculture. PFM is a crucial technological facet of agricultural production, imparting benefits such as enhanced crop yield via heightened soil temperature, retained soil moisture, and improved soil attributes (Somanathan et al., 2022; Zhang et al., 2022b). Empirical evidence establishes that PFM can potentially elevate crop yields by 20-50% (Xiong and Wu, 2022), attributed to enhanced tillage quality and reduced soil water evaporation, thereby influencing soil hydrothermal dynamics (Wen et al., 2023). FM’s implementation is linked to notable reductions in soil pond hydrogenase and bulk density, coupled with augmented surface soil organic matter (Smets et al., 2008; Mi et al., 2021). FM’s positive impact on plant nutrient absorption efficiency results in heightened yield and metabolite accumulation (Martins et al., 2021). Furthermore, investigations indicate that FM can lead to diminished SOC and soil TN levels in the 0-30 cm soil layer (Gu et al., 2019), attributed to mulching accelerating the nitrogen mineralization process in soil, unfavorably influencing soil organic carbon storage. The effects of FM on soil properties vary due to distinct plant characteristics, regional environments, and soil quality conditions.

Drip irrigation, recognized as a potent water-saving technique, operates by delivering water and nutrients directly to the plant’s root zone (Patanè and Cosentino, 2010). This method curbs water wastage, amplifies water utilization efficiency (Rodrigues Pinheiro et al., 2019), and contributes to sustaining consistent crop yield or quality (Alordzinu et al., 2022; Bai et al., 2023a; Bai et al., 2023b). Studies indicate the advantages of augmenting soluble solids in crops and curbing crop yield in conditions with a 20% water reduction during irrigation (Ortega-Farias et al., 2021). In the sandy soils of China’s northwestern arid region, a moderate reduction in irrigation proves beneficial, enhancing starch content and vitamin C in fruits (Wang et al., 2011), while simultaneously boosting yield (Li et al., 2023).

Mild water deficit (maintaining 60-65% of soil volume water content) heightens soluble matter and soluble sugar content during fruit growth and maturation (He et al., 2023), whereas reduced water irrigation (soil parent material potential of -25 kPa) bolsters potato tuber yield at initial and expansion stages (Xing et al., 2022). Therefore, integrating deficit irrigation with FM not only efficiently enhances soil moisture and nutrient retention but also substantially contributes to water conservation. Crop yield and quality stand influenced by management practices such as drip irrigation and FM (Biswas et al., 2015; Yavuz et al., 2021). Consequently, comprehending the drivers of production and quality alteration assumes paramount importance. A multitude of factors wield sway over the intricate interplay between soil-plant attributes and yield, encompassing aspects like moisture, temperature, and nutrients, among others (Huang et al., 2017; Lu et al., 2021; Ng et al., 2023). In a broader sense, SWC can serve as a pivotal element for elucidating shifts in crop yield (Daryanto et al., 2017; Alves Rodrigues Pinheiro and Nunes, 2023). Nevertheless, the actual nutrient concentration in soil and leaves significantly impacts the outcome (Bhat et al., 2012). For instance, Sawchik and Mallarino (2008) underscored that soybean yield was constrained by the availability of phosphorus and potassium. Similar investigations by Santi et al. scrutinized the influence of soil chemistry and physical parameters on soybean yield, identifying soil potassium content and infiltration rate as primary factors impinging on crop productivity (Santi et al., 2012). Nonetheless, certain reports have documented inverse associations between crop yield and soil salt, pH, and leaf nitrogen (Casanova et al., 1999; Bhat et al., 2012), while manifesting positive correlations with soil respiration, soil organic matter, and leaf calcium (Bhat et al., 2012; Gupta and Germida, 2015).

In addition to yield, crop quality indicators such as ether extract, crude protein and soluble solids are influenced by soil conditions and crop characteristics (Johansson et al., 2020). Crude protein and crude fat are important indicators for the evaluation of crop quality and for the determination of yield (Chen et al., 2022) and there is a correlation between soil conditions and these indicators. Previous studies showed significant correlations between potato crude protein and soil electrical conductivity (EC) and potassium (K) (Xing et al., 2022). Nadeem Shah et al. (Nadeem Shah et al., 2023) suggested that soil moisture and plant protein are significantly and positively correlated, and that decreasing moisture reduces maize protein content. However, Hlisnikovský et al. (2020) found that moisture deficit actually increased crude protein content in winter wheat. Soil environmental changes also affect soluble solids (Johansson et al., 2020). Previous studies have demonstrated a negative correlation between soluble solids and soil pH, as well as leaf phosphorus content (Liu et al., 2022). Lobos et al. and Ortega-Farias et al. (Lobos et al., 2018; Ortega-Farias et al., 2021) found inverse correlations between soil moisture and tuber soluble starch, in particular, moderate water deficit increased starch and soluble sugar content, which is consistent with the results of our study. However, other studies showed that tuber starch increased with increasing soil moisture (Wszelaczyńska et al., 2015) or had no effect (Zhang et al., 20-22). Rightly, Xing et al. (2022) reported a positive relationship between starch content and factors including soil water content (SWC), pH and soil available potassium (AK), but a negative relationship between starch content and leaf nitrogen.

Previous studies have shown that various factors such as crop species, local environment, soil conditions and so on are interacting with each other to limit the formation of crop yield and crop quality. Particularly in arid and semi-arid areas, there is a need for further research into the key factors that have a clear impact on the formation of crop yield and quality. The arid northwestern region,especially the southern part of southern Xinjiang, experiences a parched climate, meager rainfall, substantial evapotranspiration, and a pronounced water deficit (Chongyi et al., 2009). Consequently, plastic film mulch and drip irrigation technologies have gained extensive adoption in this area. Water-saving practices hold the potential to enhance soil conditions, thereby augmenting both crop yield and quality. Nevertheless, the specific impact of soil water scarcity on crop yield and quality under plastic film coverage demands further investigation. The crop *Cyperus esculentus*, characterized by clumped stems and leaves, robust tillering, a propensity for light, drought resistance, and tolerance to desiccation (Zhang et al., 2022a), serves as a noteworthy example. Notably, the aboveground stems and leaves of *C. esculentus* find application as fodder, while the subterranean tubers serve purposes ranging from oil extraction to brewing and beverages, and even soil and sand stabilization due to its developed root system (Bezerra et al., 2023). The versatility of *C. esculentus* in terms of food, oil, grazing, and fodder renders it both ecologically and economically valuable. Hence, the pursuit of enhanced yield and quality in *C. esculentus* holds significant promise. Consequently, the present study has selected *C. esculentus* as the focal subject to investigate the impact of water deficit under FM treatment. The objectives encompass (1) discerning the ramifications of deficit irrigation and FM on the yield and quality of *C. esculentus*, and (2) identifying the key drivers that underpin yield and quality fluctuations in *C. esculentus*.

## 2 Materials and methods

### 2.1 Studying area

**Figure 1:**
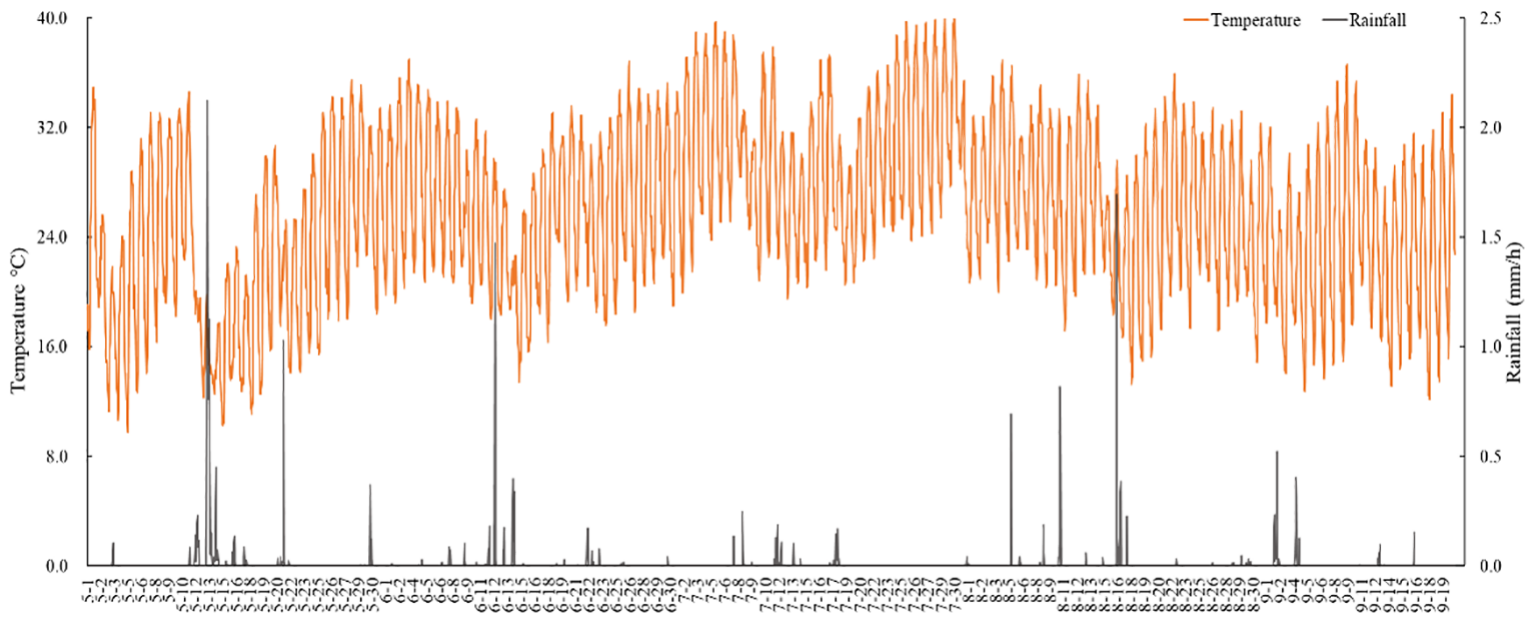
Daily average rainfall and temperature in the study area.

The field experiments were conducted at the Hala Yugong Township, *C. esculentus* planting demonstration zone in Korla, Xinjiang, which is located at approximately 41°36′N, 83°3′E. The region experiences a typical continental temperate climate. The average daily temperature during the reproductive period of *C. esculentus* in this area are around 25.76°C, and average daily rainfall 0.0095 mm (**Figure 1**). The annual evaporation rate is 2788.2 mm. The region enjoys an average of 3045 hours of sunshine per year. There are also more than 180 days without frost. In addition, the groundwater table is at a depth of 14 meters. The soil type is sandy loam, and the characteristics of the 0-40 cm soil are: the field capacity is 18%, the soil bulk density is 1.58 g/cm^3^, and soil organ matter is 0.459%.

### 2.2 Experimental design

**Figure 2:**
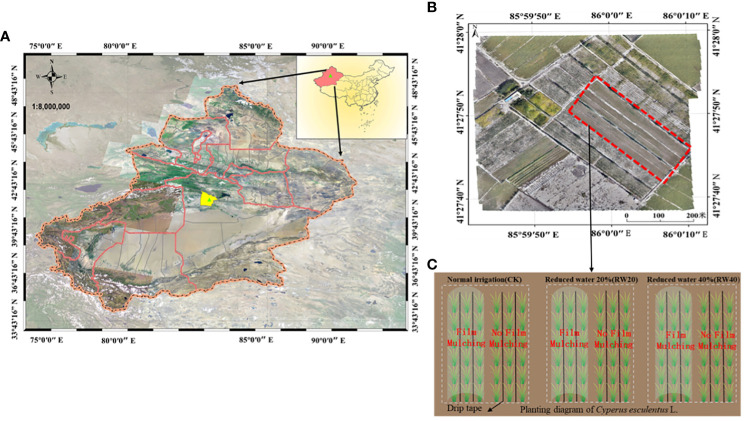
Distribution of research area and experimental design: **(A)** indicates the research area; **(B)** indicates the specific experimental area; **(C)** indicates the design of the field experiment.

The randomized block split-plot design. The experimental setup included two factors: mulchin treatment includes film mulching (FM) and no film mulching (NFM), and deficit irrigation (DI) treatments include: CK is the commonly used drip irrigation system in the local area; RW20, reducing water by 20% and RW40, reducing water by 40% (**Figure 2**). Thus, the study therefore consisted of six treatments, each with four replicates plots and plot size of 15 m^2^(3 cm×5 cm). The sowing amount is 75 kg/ha and the density of plantations is about 17000 plants/ha. Plant spacing was 12.5 cm and row spacing was 30 cm. The water dripping was controlled by water meter. The fertilizer was dissolved in water and delivered directly to the root system of *C. esculentus* drip irrigation. All treatments have the same fertilization amount: urea 75 kg/ha and bacterial fertilizer 105 kg/ha.

### 2.3 Sample Collection

Four sample areas (100 cm × 100 cm) per treatment were randomly selected as replicates at the peak of *C. esculentus* growth, starting in October 2020. Harvesting of the aboveground and underground parts of the plants (1 m×1 m) was done and was taken back to the laboratory for drying at a constant temperature of 65°C for 48h. The dry weight of both grass and tubers was measured, and the yield of both grass and tubers was also determined. The surface litter was removed, and soil samples were collected from 0-30 cm soil depth with a 10 cm diameter soil drill. The plant roots and stones were removed and samples were mixed evenly for the determination of soil physicochemical properties.

### 2.4 Determination of nutrient and quality content

The total carbon (TC) was analyzed using the K_2_Cr_2_O_7_ oxidation method. The total nitrogen (TN) was determined using an elemental analyzer (K1160, Jinan Hanon Instruments Co. Ltd., China). After wet digestion with HClO_4_-H_2_SO_4_ (iCAP 6300, Thermo Elemental, USA), using a UV spectrophotometer the total phosphorus (TP) was measured. For determining the available nitrogen (AN), the alkaline hydrolysis method was employed. To extract the available phosphorus (AP), the ascorbic acid/molybdate method was utilized and quantified through a continuous-flow autoanalyzer using colorimetric measurements. The soil moisture content was measured after drying (SWC) and soil bulk density (SBD) was measured by the ring knife method. Soil electrical conductivity (EC) and pH were determined by preparing mixtures with soil-to-nanopure water ratios of 1:5 (w/v) and 1:2.5 (w/v). The pH measurements were performed using a pH meter (Precision and Scientific Corp., China).

The ether extract was determined by hydrolysis according to the Association of Official Analytical Chemists (AOAC) method 954.02 (2006). The crude protein was determined through the Coomassie Blue G250 staining method (Grubben et al., 2019). Total soluble sugars were determined by the Anthrone colorimetric method (Rudack et al., 2017). The starch content was determined by iodine colorimetry (Duan et al., 2012).

### 2.5 Data analysis

We used SPSS 21.0 (SPSS Inc., Chicago-IL, U.S.A.) to perform normality tests, variance analyses, and multiple comparisons. The Shapiro-Wilk normality test was used to evaluate the normality of the original data. Two-way anova was used to determine the effects of plastic film mulch, irrigation and their interactions on plant and soil nutrition, yield, quality, and comparing means ± standard errors (SE) by Duncan’s method (α = 0.05). The “ggplot” package (Wilkinson, 2011) in R version 4.0.3 (R Core Team, 2020) is used for plotting, and the “random forest” package was used to construct a model to explore the key factors affecting yield and quality.

## 3 Results

### 3.1 Soil Property parameters

**Table 1** shows the significant effects of the drip irrigation (DI) on the TP, AP and EC of the soil; soil OC was significantly affected by the mulchin treatment (MT); while DI×MT interaction significantly affected soil TN, AN, SWC, pH, BD and SOM contents (**Table 1**). Specifically, the water reduction treatment led to a substantial increase in soil TN, TP, AN, and AP contents when compared to the CK treatment, regardless of the mulchin treatment (**Table 2**). Notably, the RW20 treatment within the FM conditions showcased the highest values of soil TN, TP, AN, and AP contents across all treatments. Similarly, both the CK and RW20 treatments exhibited elevated levels of soi OC and OM contents compared to RW40 under the mulchin treatment (**Table 2**). Whether FM or NFM, the CK treatment prominently raised soil SWC, EC, and BD. Interestingly, only the RW40 treatment exhibited a significant increase in soil pH, regardless of the presence of film mulching.

**Table 1:** Two-way ANOVA on the effects of mulchin treatment (MT) and deficit irrigation (DI) treatments on soil physical and chemical properties.

Treatment	SOC (g/kg)	STN (g/kg)	STP (g/kg)	AN (mg/kg)	AP (mg/kg)	SWC	pH	EC (S/m)	BD (g/cm^3^)	SOM (g/kg)
DI	2.82	19.095***	13.1***	29.09***	31.1***	686.577***	48.599***	9.439***	5.518**	13.759***
MT	45.361**	27.674***	3.89	27.674***	3.89	246.154***	8.3333**	1.95	10.588**	2.41
DI×MT	1.47	63.848***	0.17	63.848***	0.17	15.500***	3.911**	0.17	7.729**	11.801***

*, **, and *** correspond to F value were significant at P ≤ 0.05, ≤ 0.01, and ≤ 0.001, respectively. Soil organic carbon (SOC), soil total nitrogen (STN), soil total phosphorus (STP), soil available nitrogen (SAN), soil available phosphorus (SAP), soil water content (SWC), pondus hydrogenic (pH), electrical conductance (EC), soil bulk density (SBD), and soil organic matter (SOM).

**Table 2:** Effect of mulchin treatment (MT) and deficit irrigation (DI) treatments on soil nutrients.

DI	MT	SOC (g/kg)	STN (g/kg)	STP (g/kg)	AN (mg/kg)	AP (mg/kg)	SWC	pH	EC (S/m)	BD(g/cm^3^)	SOM (g/kg)
CK	NFM	2.67±0.06	0.31±0.01B	0.59±0.03B	21.84±0.61B	9.8±0.50B	0.35±0.01Ab	7.46±0.01A	0.25±0.02A	1.6±0.04A	4.6±0.19A
	FM	2.28±0.15	0.31±0.01B	0.55±0.02B	22.07±0.87B	9.16±0.34B	0.41±0.01Aa	7.34±0.02A	0.28±0.04A	1.6±0.03A	5.23±0.16A
RW20	NFM	3.08±0.12a	0.54±0.02Aa	0.72±0.02A	38.58±1.74Aa	11.98±0.33A	0.20±0.01Bb	7.52±0.01B	0.22±0.00B	1.56±0.00B	5.23±0.17A
	FM	2.33±0.14b	0.54±0.03Aa	0.66±0.03A	17.91±0.76Ab	11.05±0.44A	0.33±0.01Ba	7.54±0.02B	0.23±0.01B	1.57±0.01B	4.43±0.24A
RW40	NFM	2.82±0.05a	0.54±0.04Ba	0.51±0.02C	28.89±0.57Ba	8.58±0.34C	0.09±0.01Cb	7.86±0.08C	0.17±0.01C	1.55±0.01A	3.58±0.11B
	FM	2.13±0.12b	0.54±0.05Ba	0.49±0.03C	15.34±0.49Bb	8.13±0.53C	0.16±0.01Ca	7.68±0.04C	0.19±0.01C	1.64±0.01A	4.47±0.23B

Mulchin treatment: no film mulching (NFM), film mulching (FM). Water treatments: normal irrigation (CK), reduced by 20% water (RW20), reduced by 40% water (RW40). Mean ±SE (n=4). Duncan’s test was used to determine differences (P<0.05) between all treatments. We used lowercase letters to signify Differences between MT treatments under the same DI treatments. Soil organic carbon (SOC), soil total nitrogen (STN), soil total phosphorus (STP), soil available nitrogen (SAN), soil available phosphorus (SAP), soil water content (SWC), pondus hydrogenic (pH), electrical conductance (EC), soil bulk density (SBD), and soil organic matter (SOM).

### 3.2 The stoichiometry characteristics of leaves, roots, and tubers

**Figure 3:**
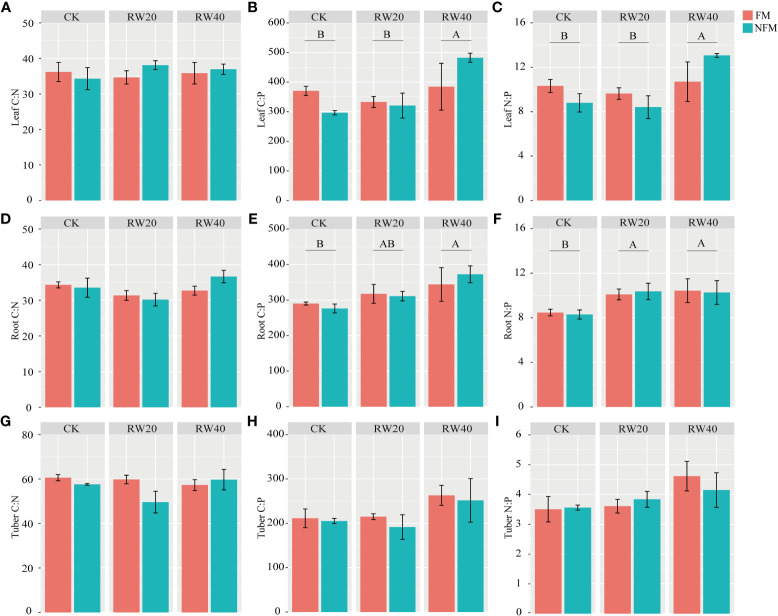
Characteristics of stoichiometric ratios of different organs mulchin treatment (MT) and deficit irrigation (DI) treatment. **(A-C, D-F, G-I)** indicates the stoichiometric ratios of nutrients in leaves, roots and tubers respectively. MT treatment includes film mulching (FM) and no film mulching (NFM) treatment. DI treatments: normal irrigation (CK), reduced by 20% water (RW20), reduced by 40% water (RW40). Total carbon (TC); Total nitrogen (TN); Total phosphorus(P). Duncan’s test to determine differences (*P*<0.05) between all treatments. To use capital letters to signify differences between drip irrigation treatments; use lowercase letters to signify differences between mulchin treatments.

The **Table 3** observed no significant impact from MT, DI treatment, or their interaction on the indicators of leaf, root, and tuber stoichiometry. Specifically, the C: N ratio of leaves and roots, as well as the C: P and N: P ratios in tubers, were measured (**Figure 3**). However, the DI treatment, excluding the PF treatment and interaction treatments, exhibited a notable influence on the C: P and N: P ratios of leaves and roots in *C. esculentus*. It is worth mentioning that the C: N ratio of tubers was not considered in the analysis. Interestingly, the stoichiometric values of leaves, roots, and tubers were higher under the RW40 treatment compared to other treatments, indicating that a reduction in water content can result in increased C: P and N: P ratios in tubers.

**Table 3:** Two-way ANOVA on the effects of mulchin treatment (MT) and deficit irrigation (DI) treatments on on plant nutrient properties.

Treatment	Leaf (L)			Root (R)			Tuber (Tu)		
	C:N	C:P	N:P	C:N	C:P	N:P	C:N	C:P	N:P
DI	0.157	4.793**	5.01**	2.95	4.422**	4.433**	1.206	2.556	2.664
MT	0.214	0.016	0.027	0.228	0.019	0.002	2.034	0.399	0.037
DI×MT	0.641	2.55	2.525	1.385	0.403	0.059	2.12	0.054	0.444

*, **, and *** correspond to F value were significant at P ≤ 0.05, ≤ 0.01, and ≤ 0.001,respectively.Total carbon (TC); Total nitrogen (TN); Total phosphorus (TP).

### 3.3 Characteristics of yield and biomass allocation

**Figure 4:**
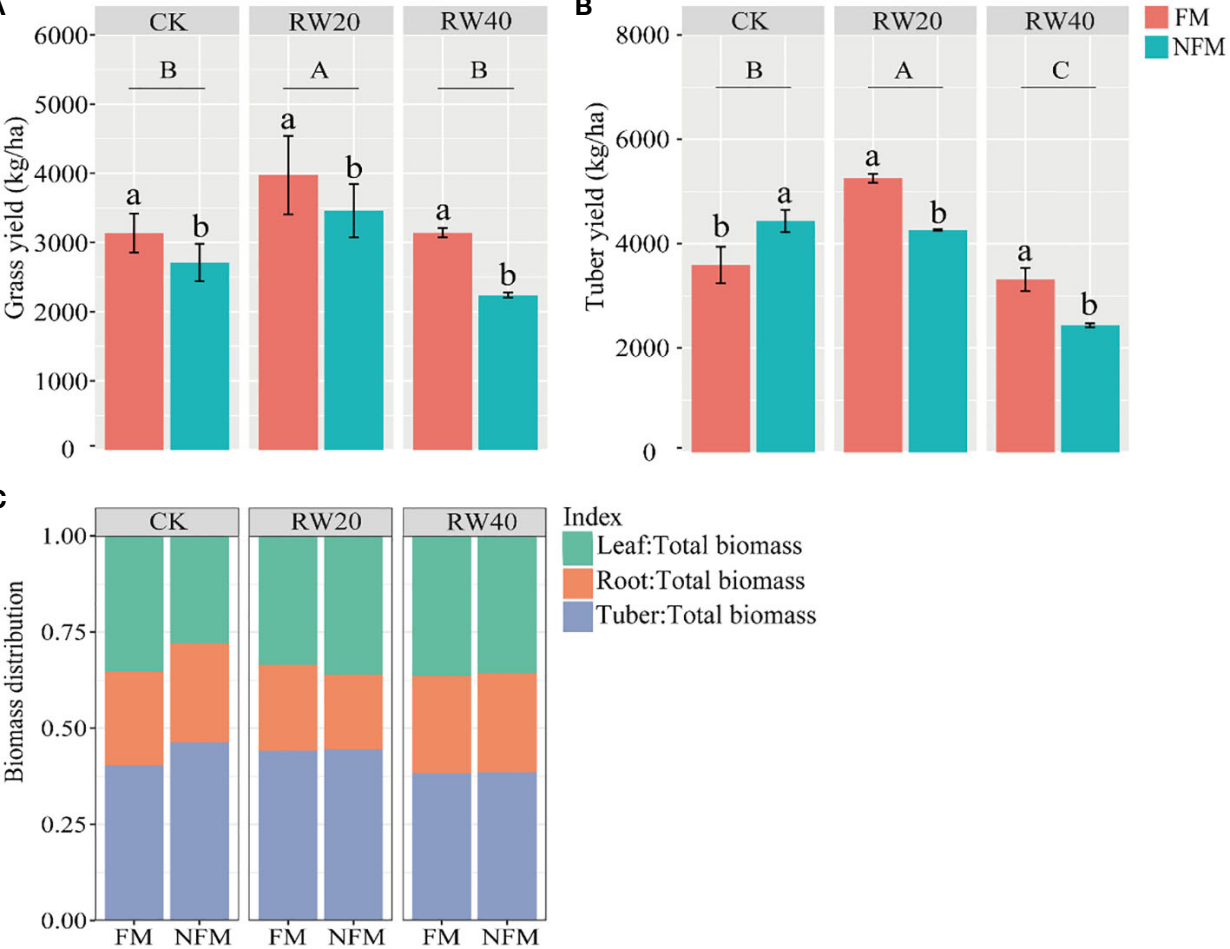
Yield and biomass distribution characteristics under mulchin treatment (MT) and deficit irrigation (DI) treatment. **(A, B)** indicate the yields of grass and tuber under different treatments; **(C)** indicates the proportion of leaf, root and tuber quality. MT treatment includes film mulching (FM) and no film mulching (NFM) treatment. DI treatments: normal irrigation (CK), reduced by 20% water (RW20), reduced by 40% water (RW40). Leaf, leaf biomass, Root, root biomass, Tuber, tuber biomass. Duncan’s test to determine differences (*P*<0.05) between all treatments. To use capital letters to signify differences between drip irrigation treatments; use lowercase letters to signify differences between mulchin treatments.

The result showed that MT treatment, DI treatment, and their combination significantly affected the yield of grass and tuber (**Table 4**). Under film mulching treatment, grass and tuber yields initially increased and then decreased with the reduction in water (**Figures 4A, B**). The highest yield of grass and tuber was obtained with RW20 treatment. Compared with NFM treatment, FM treatment significantly increased grass and tuber yield by 17.99% and 8.46%, respectively.

**Table 4:** Two-way ANOVA on the effects of mulchin treatment (MT) and deficit irrigation (DI) treatment on biomass properties.

Treatment	Biomass allocation			Yield	
	leaf	root	tuber	Grass yield (GY, kg/ha)	Tuber yield (TuY, kg/ha)
DI	4.113*	4.545*	7.294***	5.514**	48.054***
MT	2.331	0.103	2.118	5.393*	4.690*
DI×MT	4.860*	0.891	2.118	0.305**	14.115***

*, **, and *** correspond to F value were significant at P ≤ 0.05, ≤ 0.01, and ≤ 0.001, respectively.

Deficit irrigation treatment, the interaction between MT and DI treatments had a significant effect on the distribution of the leaves (**Table 4**). While roots and tubers distribution were significantly affected by the deficit irrigation treatment (**Figure 4C**). Under FM treatment, the distribution of biomass among leaves, roots, and tubers was unaffected significantly by the various deficit irrigation treatments. While reducing water significantly decreased leaf biomass distribution, and increased the tuber biomass distribution. At the same CK treatment condition, the biomass distribution of leaves under FM treatment significantly increased by 2.78% compared with no FM treatment.

### 3.4 Quality characteristics of grass and tubers

**Figure 5:**
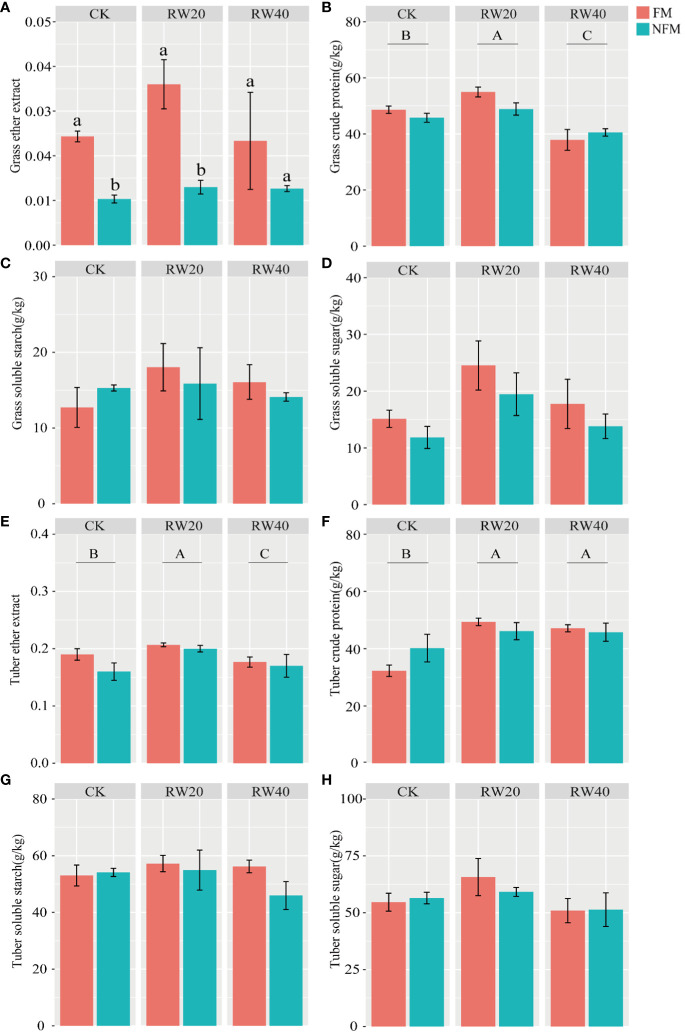
Quality characteristics of grass and tuber under mulchin treatment (MT) and deficit irrigation (DI) treatment. **(A–D)** indicate the ether extract, crude protein, soluble starch ,soluble sugar of grass; fig **E–H** indicate the ether extract, crude protein, soluble starch, soluble sugar of tuber. MT treatment includes film mulching (FM) and no film mulching (NFM) treatment. DI treatments: normal irrigation (CK), reduced by 20% water (RW20), reduced by 40% water (RW40). Duncan’s test to determine differences (P<0.05) between all treatments. To use capital letters to signify differences between drip irrigation treatments; use lowercase letters to signify differences between mulchin treatments.

We found that grass ether extract and crude protein contents were significantly affected by plastic film mulching and deficit irrigation treatment (**Table 5**). The amount of water regime was found to considerably influence the ether extract and crude protein contents of tubers (**Table 5**). First, FM treatment significantly improved ether extract and crude protein accumulation in the grass. Under FM treatment, the ether extract, crude protein, soluble starch, and soluble sugar contents of grass initially increased and then decreased as irrigated water was decreased (**Figures 5A–D**). On the other hand, the RW40 and CK treatments also displayed reduced ether extract, crude protein, soluble starch, and soluble sugar contents of grass under NFM conditions.

**Table 5:** Two-way ANOVA of effects of mulchin treatment (MT) and deficit irrigation (DI) treatment on grass and tuber quality.

Treatment	Leaf				Tuber			
	ether extract (EE, %) %	crude protein (CP, g/kg)	soluble starch (SS, g/kg)	soluble sugar (ss, g/kg)	ether extract (EE, %)	crude protein (CP, g/kg)	soluble starch (SS, g/kg)	soluble sugar (ss, g/kg)
DI	1.226	17.844***	0.594	3.729	3.987*	9.631***	0.725	2.208
MT	14.807***	1.412	0.055	2.421	2.195	0.218	1.261	0.109
DI×MT	0.796	2.105	0.482	0.039	0.636	2.150	0.983	0.339

*, **, and *** correspond to F value were significant at P ≤ 0.05, ≤ 0.01, and ≤ 0.001, respectively.

Similar to the variation in the quality of the grass. The ether extract, crude protein, soluble starch, and soluble sugar contents of tubers increased first and then decreased with decreasing water under FM conditions (**Figures 5E–H**). The RW40 and CK treatments also exhibited decreased ether extract, crude protein, soluble starch, and soluble sugar contents of tubers under the NFM treatment. Based on the above analysis, 20% water reduction promoted the accumulation of ether extract, crude protein, soluble starch, and soluble sugar contents in both *C. esculentus* leaves and tubers.

### 3.5 Predictors of quality and yield of *C. esculentus*

**Figure 6:**
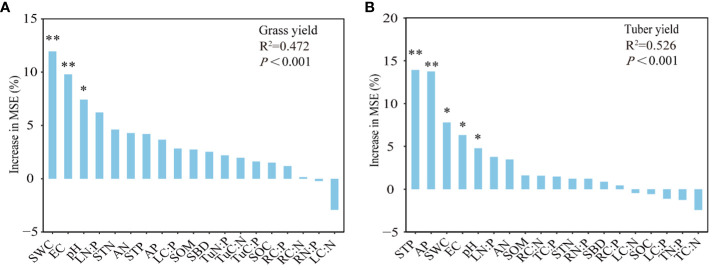
Prediction factors for yield of *C. esculentus*. **(A, B)** indicate the main environmental factors that affect the yield of grass and tuber, respectively. Grass yield (GY), tuber yield (TuY). Leaf (L), root (R), tuber (Tu). Soil organic carbon (SOC), total nitrogen (TN), total phosphorus (TP), soil available nitrogen (SAN), soil available phosphorus (SAP), soil water content (SWC), pondus hydrogenic (pH), electrical conductance (EC), soil bulk density (SBD), and soil organic matter (SOM). Indicated significant levels *, **correspond to *P*-value of ≤ 0.05, ≤ 0.01, respectively.

**Figure 6A** showed that the prediction factors in yield of *C. esculentus*. Soil SWC, EC, and pH significantly influenced the variation in grass yield, thus soil SWC, EC, pH, TP, and AP contents significantly influenced the variation in tuber yield (**Figure 6B**).

**Figure 7:**
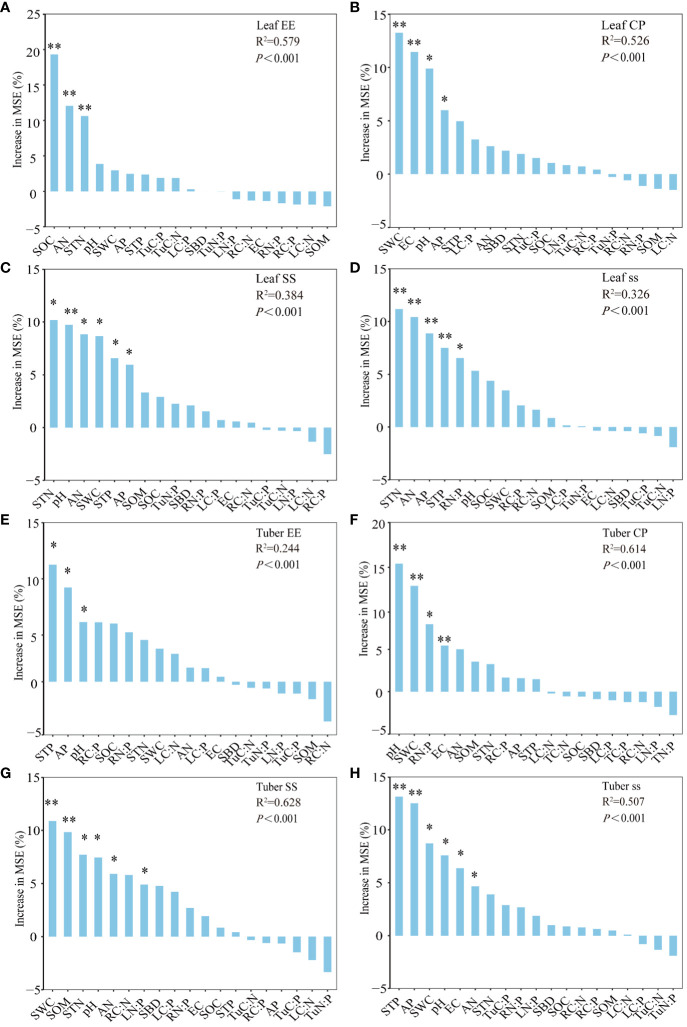
Prediction factors for quality of *C. esculentus*. **(A–D)** indicate the main environmental factors influencing grass quality; **(E–H)** indicate the main environmental factors influencing tuber quality. Grass ether extract (GEE), grass crude protein (GCP), grass soluble starch (GSS), and grass soluble sugar (Gss). Tuber ether extract (TuEE), tuber crude protein (TuCP), tuber soluble starch (TuSS), and tuber soluble sugar (Tuss). Leaf (L), root (R), tuber (Tu). Soil organic carbon (SOC), total nitrogen (TN), total phosphorus (TP), soil available nitrogen (SAN), soil available phosphorus (SAP), soil water content (SWC), pondus hydrogenic (pH), electrical conductance (EC), soil bulk density (SBD), and soil organic matter (SOM). Indicated significant levels *, **correspond to *P*-value of ≤ 0.05, ≤ 0.01, respectively.

In the quality of grass and tubers of *C. esculentus* (**Figures 7A–H**). Soil TN, AN, and OC contents significantly influenced the variation in grass ether extract (**Figure 7A**). The variation in grass crude protein content was significantly influenced by the dominant factors of soil AP contents, SWC, pH, and EC (**Figure 7B**). The soluble starch of grass contents variation was significantly impacted by soil TN, AN, TP, and AP contents, SWC, and pH (**Figure 7C**), However, soil TN, AN, TP, AP contents, and RN: P ratio contributed the most to soluble sugars in grass (**Figure 7D**). Different patterns of predictive factors for tuber and grass quality. Soil STP, AP, and pH contribute the most to the ether extract content of tubers (**Figure 7E**). The main factors significantly regulating and impacting tuber crude protein content variation were soil SWC, pH, EC, and RN: P ratio (**Figure 7F**). Soil TN, AN, OC contents, SOM contents, SWC, and pH, contribute the most to tuber soluble starch (**Figure 7G**). Soil AN, TP, AP contents, SWC, pH, and EC, contributed the most to the soluble sugar of tubers (**Figure 7H**).

## 4 Discussion

### 4.1 Effect of irrigation and film mulching on *C. esculentus* yield and quality

The yield of crops is influenced by a myriad of factors, including water content (Kirda et al., 2004), management practices such as irrigation, FM, and fertilization (Bai et al., 2022), soil characteristics (Sheng et al., 2023), and plant-specific attributes such as nutrients and leaf area index (Puccinelli et al., 2023). In our study, the interplay of DI, MT, and their interaction significantly impacted the yields of both grass and tubers. under the condition of a 20% reduction in water supply, the average yield of grass and tubers increased significantly by 6.31% and 23.13% respectively, compared to the CK treatment (**Figure 4A**). Bhattacharyya et al. (2018) Study on maize in an inceptisol of West Bengal, India found that 50% available soil moisture deficit (CK:75% available soil moisture deficit) significantly increased yield, which is consistent with the results of our study. However, some studies have shown that regulated deficit irrigation can reduce crop yield (Papastylianou and Argyrokastritis, 2014), depending on the irrigation period (McCarthy, 2005). For instance, water deficit during the flowering and seed filling stages of rice significantly reduces rice seed yield (Vijayaraghavareddy et al., 2020). It has also been shown that water deficit (20-40%) reduces wheat yield less (less than 25%) (De Santis et al., 2021). This may depend on the crop species, the genetic evolution of the species during long-term domestication of the crop, and the progressive adaptation to changes in the agricultural environments (Ahmed et al., 2020).

Plastic film mulch has been used as an agricultural cover to increase crop yield (Thakur and Kumar, 2021). Related studies have shown that mulching increases the yield of various types of crops, with mulching increasing the yield of seed maize > potato > maize > cotton > soybean compared to no mulching (Bandyopadhyay et al., 2023; Dewi et al., 2024). This study showed that the yield of mulched grass and tuber was 17.99% and 10.60% higher than unmulched, respectively, which is consistent with previous studies. Black mulches increase soil temperature and adversely affect plant survival and photosynthesis, resulting in low yield (Bhardwaj, 2013). Differences in the colour of the mulch also have some effect on the variability of the yield (Thakur and Kumar, 2021). The study of Sarrou showed that the grass yield of Ocimum basilicum L. under black mulch cover (19900.099 kg/ha) was significantly higher than that of bare soil (Sarrou and Chatzopoulou, 2016). Similar results were reported in Abaas’ study of marigold (Abaas, 2014). On the other hand, Thankamani’s study on ginger showed that white, cream-coloured mulch was more productive than black mulch (Thankamani et al., 2016). In contrast, Hanna McIntosh’s study reported that black, white and gold plastic films all increased raspberry yield, and black plastic film increased yield more than white and gold films (McIntosh et al., 2023). It is widely acknowledged that most inorganic mulches are non-biodegradable. Non-biodegradable plastics are also associated with pollution problems, such as microplastics (Sintim et al., 2022). New biodegradable plastic mulches have been developed (Sintim et al., 2022; Dewi et al., 2024) that have the potential to increase moisture content, soil temperature, prevent soil pathogens, and reduce weed growth. These films can be degraded relatively quickly (Bianchini et al., 2022; Tsuboi et al., 2022). However, further research is needed to fully explore the benefits of these biodegradable films in agriculture.

In the realm of agricultural production, indicators of crop quality such as ether extract, crude protein, and total soluble solids serve as critical benchmarks for evaluating field management and overall crop quality (Gomiero, 2018). The research underscores that the nutritional value of crops hinges on the content of various nutrients, with higher levels of ether extract and crude protein correlating with elevated nutritional value (Chen et al., 2013). Our study unveiled noteworthy impacts, as mulching significantly affected the crude fat content of grass. Furthermore, grass crude protein, tuber crude fat, and crude protein were substantially influenced by variations in irrigation amounts. Our findings align with similar research that highlights the influence of agricultural practices like FM and drip irrigation on changes in crude fat and protein content of crops (Biswas et al., 2015; Bai et al., 2022). The observed trends indicated that with diminishing irrigation, the average crude fat and protein content of both grass and tubers exhibited an initial increase followed by a subsequent decline. This pattern resonates with the research conducted by Jiang et al. (2022) on the quality *of Avena sativa* L. However, this trend diverges from the conclusion drawn by Kou et al. (2014), which suggests that the crude protein content of *alfalfa* gradually rises as water content increases. This inconsistency may be attributed to the unique biological traits of *C. esculentu*s, encompassing growth patterns, root structure, and physiological adaptations, which can influence its response to agricultural interventions. Additionally, the interplay of climate, light, temperature, and soil types within the study area can profoundly impact the effectiveness of irrigation and mulching practices. Similarly, the soil type specific to the study area emerges as a pivotal factor influencing the intricate relationship between DI, FM, and *C. esculentus*. Therefore, the aim of future research should be the investigation of the intrinsic relationship between plants and the environment.

### 4.2 Factors affecting the yield and quality of *C. esculentus*

Irrigation and management practices wield substantial influence over soil physical attributes, nutrient content, and nutrient uptake in plants (Amer, 2010; Wang et al., 2011). Soil-plant mineral nutrients are considered important indicators for assessing crop nutritional status, nutritional value, yield relationship and quality attributes (Casanova et al., 1999). Relevant studies have shown that the nitrogen and potassium content of the leaves are the most important factors influencing the crop yield (Singh et al., 2022). In contrast, Korkmaz et al (Korkmaz et al., 2015). found that nitrogen and Mn in plant tissue played a significant role in potato yield and nodulation. Meanwhile, Sawchik’s study on soybean indicated that plant phosphorus and potassium efficacy significantly affected soybean yield in both fields (Sawchik and Mallarino, 2008). In our study, SWC, EC and PH were the main factors influencing grass yield, while tuber yield was influenced by STP, AP, SWC, EC and PH respectively, so it can be seen that different crop types have different responses to the environment. our research substantiates the close association between crop yield and pH and EC, reinforcing the importance of soil SWC, pH, and EC in influencing grass and tuber yields within this region.

Beyond yield, quality indicators also exhibit sensitivity to soil and crop characteristics. The random forest results indicate that reveal the significant influence of soil SWC, TN, and AN contents on grass ether extract content. Likewise, STP, AP content, and pH emerge as principal determinants of tuber ether extract content, suggesting the nuanced impact of management practices (irrigation and film mulching) on soil’s physical and biological properties. Grass crude protein content is primarily influenced by soil SWC, pH, EC, and soil AP content, whereas soil SWC, pH, EC, and root N:P ratio significantly affect tuber crude protein content changes. EC and pH are vital indicators for water-soluble salt levels in soils, essential for understanding soil suitability for plant growth and recognizing potential constraints imposed by saltions (Bhardwaj et al., 2019; Lei et al., 2021). Furthermore, soil SWC, AP content, and root N:P ratio also emerge as pivotal factors impacting grass and tuber crude protein contents. This may be because plants increase protein catabolism in response to reduced soil moisture to satisfy energy and ammonia reserves, while suppressing protein energy (Singh et al., 2021; Hazrati et al., 2022). These changes are adaptive strategies used by plants to respond to stress and help maintain essential metabolic and survival functions (Costa et al., 2007).

Besides crude proteins and ether extracts, non-structural carbohydrates are involved in material partitioning, yield and quality formation as the main products of plant photosynthesis. Research by Yang et al. highlighted the role of soil moisture in solute storage across plant components, emphasizing the impact of appropriate soil moisture on soluble sugar and starch accumulation (Yang et al., 2022). In our study, mulchin treatment, irrigation, and interaction insignificantly influenced soluble substances in grass and tubers. Soluble sugars and starch initially increased and then declined with reduced water availability, suggesting the optimal balance required for aboveground processes. Optimal moisture levels foster photosynthesis, enhance metabolic rates, promote dry matter accumulation, and augment yields (Calzadilla et al., 2022). Our research underscores soil TN, AN, STP, AP contents, SWC, and pH as major contributors to changes in soluble starch content in leaves, while soil TN, AN, SOC, and SOM contents, SWC, and pH, content play pivotal roles in altering soluble starch content in tubers. Soluble sugar content in leaves primarily hinges on soil TN, AN, TP, AP contents, and root N:P ratio, whereas soluble sugar content in tubers is significantly influenced by soil AN, STP, AP content, SWC, pH, and EC. While mulching and irrigation techniques indeed influence soil moisture, other factors like temperature, light intensity, and nutrient supply play equally significant roles in shaping this process. Notably, soluble starch and soluble sugar are positively correlated with yields, aligning with Ma et al. (2021) on the link between yield and soluble matter in *Chenopodium quinoa* Willd.

In conclusion, drip irrigation has the potential to be widely promoted in semiarid and arid areas to save water and increase productivity for food security. Particularly in regions with low rainfall, arid climates and high soil salinity, drip irrigation combined with mulch can significantly increase crop yields. However, practical issues such as the difficulty of installing drip irrigation systems can affect its efficiency and water quality. Insufficient foil recycling results in more foil waste, which pollutes the environment and affects soil water transport and plant growth. Farmers address these by improving irrigation and fertilisation, using biodegradable mulches, increasing mulching and adopting drip irrigation. Although trials exist, there is insufficient data to support these practices. Future research requires longer trials to assess the effects of film cover and irrigation on yield and quality.

## 5 Conclusion

Soil management practices, such as FM and watering, can change the properties of the soil, which can affect the yield, quality, and distribution of nutrients. Soil SWC, pH, and EC are key factors in regulating yield and quality. Under FM treatment, reduced 20% water treatment had the highest irrigation yield and the best quality of forage and tubers. Therefore, under the condition of film mulching in this area, reducing water by 20% is an irrigation management strategy to achieve water-saving goals and achieve high yield.

## Data availability statement

The original contributions presented in the study are included in the article/**Supplementary Material**. Further inquiries can be directed to the corresponding authors.

## Author contributions

YD: Conceptualization, Data curation, Formal analysis, Visualization, Writing – original draft, Methodology, Software, Supervision. ZZ: Methodology, Supervision, Writing – review & editing. YL: Supervision, Writing – review & editing. LL: Methodology, Writing – review & editing. WI: Methodology, Supervision, Writing – review & editing. FZ: Supervision, Writing – review & editing.

## References

[B1] Abaas I. S. (2014). Effect of biological competition of weeds on growth and volatile oil yield of marigold (Calendula officinalis L.) as medicinal plant used in Herbal medicine of Iraq. Int. J. Pharm. Pharm. Sci. 6, 217–219.

[B2] Ahmed K. Shabbir G. Ahmed M. Shah K. N. (2020). Phenotyping for drought resistance in bread wheat using physiological and biochemical traits. Sci. Total Environ. 729, 139082. doi: 10.1016/j.scitotenv.2020.139082 32371202 PMC7189857

[B3] Alordzinu K. E. Appiah S. A. Al Aasmi A. Darko R. O. Li J. Lan Y.. (2022). Evaluating the influence of deficit irrigation on fruit yield and quality indices of tomatoes grown in sandy loam and silty loam soils. Water 14, 1753. doi: 10.3390/w14111753

[B4] Alves Rodrigues Pinheiro E. Nunes M. R. (2023). Long-term agro-hydrological simulations of soil water dynamic and maize yield in a tillage chronosequence under subtropical climate conditions. Soil Tillage Res. 229, 105654. doi: 10.1016/j.still.2023.105654

[B5] Amer K. H. (2010). Corn crop response under managing different irrigation and salinity levels. Agric. Water Manage. 97, 1553–1563. doi: 10.1016/j.agwat.2010.05.010

[B6] Bai C. Zuo J. Watkins C. B. Wang Q. Liang H. Zheng Y.. (2023a). Sugar accumulation and fruit quality of tomatoes under water deficit irrigation. Postharvest Biol. Technol. 195, 112112. doi: 10.1016/j.postharvbio.2022.112112

[B7] Bai M. Tao Q. Zhang Z. Lang S. Li J. Chen D.. (2023b). Effect of drip irrigation on seed yield, seed quality and water use efficiency of Hedysarum fruticosum in the arid region of Northwest China. Agric. Water Manage. 278, 108137. doi: 10.1016/j.agwat.2023.108137

[B8] Bai Y. Zhang H. Jia S. Huang C. Zhao X. Wei H.. (2022). Plastic film mulching combined with sand tube irrigation improved yield, water use efficiency, and fruit quality of jujube in an arid desert area of Northwest China. Agric. Water Manage. 271, 107809. doi: 10.1016/j.agwat.2022.107809

[B9] Bandyopadhyay K. K. Acharya C. L. Hati K. M. (2023). Mulches and cover crops part II: Role in soil health and climate resilient agriculture. Encyclopedia Soils Environ. (Second Edition) 3, 401–413. doi: 10.1016/B978-0-12-822974-3.00228-7

[B10] Bezerra J. J. L. Feitosa B. F. Souto P. C. Pinheiro A. A. V. (2023). Cyperus esculentus L. (Cyperaceae): Agronomic aspects, food applications, ethnomedicinal uses, biological activities, phytochemistry and toxicity. Biocatalysis Agric. Biotechnol. 47, 102606. doi: 10.1016/j.bcab.2023.102606

[B11] Bhardwaj R. L. (2013). Effect of mulching on crop production under rainfed condition -a review. Agric. Rev. 34, 188. doi: 10.5958/j.0976-0741.34.3.003

[B12] Bhardwaj A. K. Mishra V. K. Singh A. K. Arora S. Srivastava S. Singh Y. P.. (2019). Soil salinity and land use-land cover interactions with soil carbon in a salt-affected irrigation canal command of Indo-Gangetic plain. Catena 180, 392–400. doi: 10.1016/j.catena.2019.05.015

[B13] Bhat R. Sujatha S. C. T. (2012). Assessing soil fertility of a laterite soil in relation to yield of arecanut (Areca catechu l.) in humid tropics of india. Geoderma 189–190, 91–97. doi: 10.1016/j.geoderma.2012.05.010

[B14] Bhattacharyya K. Das T. Ray K. Dutta S. Majumdar K. Pari A.. (2018). Yield of and nutrient-water use by maize exposed to moisture stress and K fertilizers in an inceptisol of West Bengal, India. Agric. Water Manage. 206, 31–41. doi: 10.1016/j.agwat.2018.04.038

[B15] Bianchini M. Trozzo L. D’Ottavio P. Giustozzi M. Toderi M. Ledda L.. (2022). Soil refinement accelerates in-field degradation rates of soil-biodegradable mulch films. Ital. J. Agron. 17, 2044. doi: 10.4081/ija.2022.2044

[B16] Biswas S. K. Akanda A. R. Rahman M. S. Hossain M. A. (2015). Effect of drip irrigation and mulching on yield, water-use efficiency and economics of tomato. Plant Soil Environ. 61, 97–102. doi: 10.17221/804/2014-PSE

[B17] Calzadilla P. I. Carvalho F. E. L. Gomez R. Lima Neto M. C. Signorelli S. (2022). Assessing photosynthesis in plant systems: A cornerstone to aid in the selection of resistant and productive crops. Environ. Exp. Bot. 201, 104950. doi: 10.1016/j.envexpbot.2022.104950

[B18] Casanova D. Goudriaan J. Bouma J. Epema G. F. (1999). Yield gap analysis in relation to soil properties in direct-seeded flooded rice. Geoderma 91, 191–216. doi: 10.1016/S0016-7061(99)00005-1

[B19] Chen C. Y. Wang S. W. Kim H. Pan S. Y. Fan C. Lin Y. J. (2021). Non-conventional water reuse in agriculture: A circular water economy. Water Res. 199, 117193. doi: 10.1016/j.watres.2021.117193 33971532

[B20] Chen W. Chen Y. Siddique K. H. M. Li S. (2022). Root penetration ability and plant growth in agroecosystems. Plant Physiol. Biochem. 183, 160–168. doi: 10.1016/j.plaphy.2022.04.024 35605464

[B21] Chen J. Ren X. Zhang Q. Diao X. Shen Q. (2013). Determination of protein, total carbohydrates and crude fat contents of foxtail millet using effective wavelengths in NIR spectroscopy. J. Cereal Sci. 58, 241–247. doi: 10.1016/j.jcs.2013.07.002

[B22] Chongyi E. Yong W. Taibao Y. Jiankang H. Hongchang H. Fengmei Y. (2009). Different responses Of different altitudes surrounding Taklimankan desert To global climate change. Environ. geology Water Sci. doi: 10.1007/s00254-008-1227-y

[B23] Costa J. M. Ortuño M. F. Chaves M. M. (2007). Deficit irrigation as a strategy to save water: Physiology and potential application to horticulture. J. Integr. Plant Biol. 49, 1421–1434. doi: 10.1111/j.1672-9072.2007.00556.x

[B24] Daryanto S. Wang L. Jacinthe P.-A. (2017). Global synthesis of drought effects on cereal, legume, tuber and root crops production: A review. Agric. Water Manage. 179, 18–33. doi: 10.1016/j.agwat.2016.04.022

[B25] De Santis M. A. Soccio M. Laus M. N. Flagella Z. (2021). Influence of drought and salt stress on durum wheat grain quality and composition: A review. Plants 10, 2599. doi: 10.3390/plants10122599 34961071 PMC8708103

[B26] Dewi S. K. Han Z. M. Bhat S. A. Zhang F. Wei Y. Li F. (2024). Effect of plastic mulch residue on plant growth performance and soil properties. Environ. pollut. 343, 123254. doi: 10.1016/j.envpol.2023.123254 38160772

[B27] Duan D. X. Donner E. Liu Q. Smith D. C. Ravenelle F. (2012). Potentiometric titration for determination of amylose content of starch - a comparison with colorimetric method. Food Chem. 130, 1142–1145. doi: 10.1016/j.foodchem.2011.07.138

[B28] Gomiero T. (2018). Food quality assessment in organic vs. conventional agricultural produce: Findings and issues. Appl. Soil Ecology. HUMUSICA 3 - Reviews Applications Tools 123, 714–728. doi: 10.1016/j.apsoil.2017.10.014

[B29] Grubben N. L. M. van Heeringen L. Keesman K. J. (2019). Modelling potato protein content for large-scale bulk storage facilities. Potato Res. 62, 333–344. doi: 10.1007/s11540-019-9414-7

[B30] Gu X. B. Cai H. J. Du Y. D. Li Y. N. (2019). Effects of film mulching and nitrogen fertilization on rhizosphere soil environment, root growth and nutrient uptake of winter oilseed rape in northwest China. Soil Tillage Res. 187, 194–203. doi: 10.1016/j.still.2018.12.009

[B31] Gupta V. V. S. R. Germida J. J. (2015). Soil aggregation: Influence on microbial biomass and implications for biological processes. Soil Biol. Biochem. 80, A3–A9. doi: 10.1016/j.soilbio.2014.09.002

[B32] Hazrati S. Khurizadeh S. Sadeghi A. R. (2022). Application of zeolite improves water and nitrogen use efficiency while increasing essential oil yield and quality of Salvia officinalis under water-deficit stress. Saudi J. Biol. Sci. 29, 1707–1716. doi: 10.1016/j.sjbs.2021.10.059 35280570 PMC8913395

[B33] He Z. Lu X. Cui N. Jiang S. Zheng S. Chen F.. (2023). Effect of soil water content threshold on kiwifruit quality at different growth stages with drip irrigation in the humid area of Southern China. Scientia Horticultura 307, 111477. doi: 10.1016/j.scienta.2022.111477

[B34] Hlisnikovský L. Menšík L. Kunzová E. (2020). The development of winter wheat yield and quality under different fertilizer regimes and soil-climatic conditions in the Czech Republic. Agronomy 10, 1160. doi: 10.3390/agronomy10081160

[B35] Huang L. Liu X. Wang Z. Liang Z. Wang M. Liu M.. (2017). Interactive effects of pH, EC and nitrogen on yields and nutrient absorption of rice (Oryza sativa L.). Agric. Water Manage. 194, 48–57. doi: 10.1016/j.agwat.2017.08.012

[B36] Jiang Y. B. Qi G. P. Yin M. H. Kang Y. X. (2022). Effects of water regulation and planting patterns on soil moisture,yield and quality in artificial grassland. J. Soil Water Conserv. 36, 260–270. doi: 10.13870/j.cnki.stbcxb.2022.06.032

[B37] Johansson E. Branlard G. Cuniberti M. Flagella Z. Hüsken A. Nurit E.. (2020). “Genotypic and environmental effects on wheat technological and nutritional quality,” in Wheat quality for improving processing and human health. Eds. Igrejas G. Ikeda T. M. Guzmán C. (Springer International Publishing, Cham), 171–204. doi: 10.1007/978-3-030-34163-3_8

[B38] Kirda C. Cetin M. Dasgan Y. Topcu S. Kaman H. Ekici B.. (2004). Yield response of greenhouse grown tomato to partial root drying and conventional deficit irrigation. Agric. Water Manage. 69, 191–201. doi: 10.1016/j.agwat.2004.04.008

[B39] Korkmaz K. Dede O. Erdem H. Cankaya S. Akgun M. (2015). Relationships between chemical and physical properties of soils and nutrient status of plants on yield of potato. Fresenius Environ. Bull. 24, 4108–4113.

[B40] Kou D. Su D. R. Wu D. Li Y. (2014). Effects of regulated deficit irrigation on water consumption, hay yield and quality of alfalfa under subsurface drip irrigation. Trans. Chin. Soc Agric. Eng. 30, 116–123.

[B41] Lei G. Zeng W. Jiang Y. Ao C. Wu J. Huang J. (2021). Sensitivity analysis of the SWAP (Soil-Water-Atmosphere-Plant) model under different nitrogen applications and root distributions in saline soils. Pedosphere 31, 807–821. doi: 10.1016/S1002-0160(21)60038-3

[B42] Li B. Liu Q. Yao Z. Ma Z. Li C. (2023). Mulch film: An overlooked diffuse source of organic ultraviolet absorbers in agricultural soil. Environ. pollut. 318, 120935. doi: 10.1016/j.envpol.2022.120935 36566917

[B43] Liu Z. Yang Y. Jiang X. Li S. Wu Y. (2022). Correlation of fruit quality,soil fertility and leaf nutrients of ‘Longan’pomelo. Hubei Agric. Sci. 61, 247–252. doi: 10.14088/j.cnki.issn0439-8114.2022.S1.052

[B44] Lobos T. E. Retamales J. B. Ortega-Farías S. Hanson E. J. López-Olivari R. Mora M. L. (2018). Regulated deficit irrigation effects on physiological parameters, yield, fruit quality and antioxidants of Vaccinium corymbosum plants cv. Brigitta. Irrigation Sci. 36, 49–60. doi: 10.1007/s00271-017-0564-6

[B45] Lu J. Shao G. Gao Y. Zhang K. Wei Q. Cheng J. (2021). Effects of water deficit combined with soil texture, soil bulk density and tomato variety on tomato fruit quality: A meta-analysis. Agric. Water Manage. 243, 106427. doi: 10.1016/j.agwat.2020.106427

[B46] Ma S. Liu R. Guo z. Yang R. (2021). Photosynthetic characteristics and the relationship between non-structural carbohydrates content and yield of quinoa. Jiangsu J. Agric. Sci. 37, 1378–1385.

[B47] Martins R. N. Fagundes Portes M. Fialho E Moraes H. M. Ribeiro Furtado Junior M. Fim Rosas J. T. Orlando Junior W. D. A. (2021). Influence of tillage systems on soil physical properties, spectral response and yield of the bean crop. Remote Sens. Applications: Soc. Environ. 22, 100517. doi: 10.1016/j.rsase.2021.100517

[B48] McCarthy M. G. (2005). Regulated deficit irrigation and partial rootzone drying as irrigation management techniques for grapevines. Deficit Irrig. Pract. 55t, 79–87.

[B49] McIntosh H. Guédot C. Atucha A. (2023). Plastic mulches improve yield and reduce spotted-wing drosophila in primocane raspberry. Scientia Hortic. 320, 112203. doi: 10.1016/j.scienta.2023.112203

[B50] Mi Y. W. Gong C. W. Shao W. P. Dun Z. H. (2021). Effects of plastic film mulching on soil quality,growth of Angelica sinensis,and weed occurrence in cold and humid areas. Chin. J. Appl. Ecol. 32, 3152–3158. doi: 10.13287/j.1001-9332.202109.026 34658200

[B51] Nadeem Shah M. Wright D. L. Hussain S. Koutroubas S. D. Seepaul R. George S.. (2023). Organic fertilizer sources improve the yield and quality attributes of maize (Zea mays L.) hybrids by improving soil properties and nutrient uptake under drought stress. J. King Saud Univ. - Sci. 35, 102570. doi: 10.1016/j.jksus.2023.102570

[B52] Ng C. W. W. So P. S. Coo J. L. Lau S. Y. Wong J. T. F. (2023). Interactions between nutrient types and soil hydrological properties on yield and quality of Pinellia ternata, a medicinal plant. Ind. Crops Products 195, 116423. doi: 10.1016/j.indcrop.2023.116423

[B53] Ortega-Farias S. Espinoza-Meza S. López-Olivari R. Araya-Alman M. Carrasco-Benavides M. (2021). Effects of different irrigation levels on plant water status, yield, fruit quality, and water productivity in a drip-irrigated blueberry orchard under Mediterranean conditions. Agric. Water Manage. 249, 106805. doi: 10.1016/j.agwat.2021.106805

[B54] Pan S. Y. Haddad A. Z. Kumar A. Wang S. W. (2020). Brackish water desalination using reverse osmosis and capacitive deionization at the water-energy nexus. Water Res. 183, 116064. doi: 10.1016/j.watres.2020.116064 32745671

[B55] Papastylianou P. T. Argyrokastritis I. G. (2014). Effect of limited drip irrigation regime on yield, yield components, and fiber quality of cotton under Mediterranean conditions. Agric. Water Manag. 142, 127–134. doi: 10.1016/j.agwat.2014.05.005

[B56] Patanè C. Cosentino S. L. (2010). Effects of soil water deficit on yield and quality of processing tomato under a Mediterranean climate. Agric. Water Manage. 97, 131–138. doi: 10.1016/j.agwat.2009.08.021

[B57] Puccinelli M. Carmassi G. Pardossi A. Incrocci L. (2023). Wild edible plant species grown hydroponically with crop drainage water in a Mediterranean climate: Crop yield, leaf quality, and use of water and nutrients. Agric. Water Manage. 282, 108275. doi: 10.1016/j.agwat.2023.108275

[B58] R Core Team. (2020). R: A Language and Environment for Statistical Computing. R Foundation for Statistical Computing, Vienna.

[B59] Rodrigues Pinheiro J. Lier E. A. de JongSimunek Q. (2019). The role of soil hydraulic properties in crop water use efficiency: A process-based analysis for some Brazilian scenarios. Agric. Syst. 173. doi: 10.1016/j.agsy.2019.03.019

[B60] Rudack K. Seddig S. Sprenger H. Köhl K. Uptmoor R. Ordon F. (2017). Drought stress-induced changes in starch yield and physiological traits in potato. J. Agron. Crop Sci. 203, 494–505. doi: 10.1111/jac.12224

[B61] Saha P. Sade N. Arzani A. Rubio Wilhelmi M. D. M. Coe K. M. Li B.. (2016). Effects of abiotic stress on physiological plasticity and water use of Setaria viridis (L.). Plant Sci. 251, 128–138. doi: 10.1016/j.plantsci.2016.06.011 27593471

[B62] Santi A. L. Amado T. J. C. Cherubin M. R. Martin T. N. Pires J. L. Flora L. P. D.. (2012). Análise de componentes principais de atributos químicos e físicos do solo limitantes à produtividade de grãos. Pesqui. Agropecu. Bras. 47, 1346–1357. doi: 10.1590/S0100-204X2012000900020

[B63] Sarrou E. Martens S. Chatzopoulou P. (2016). Metabolite profiling and antioxidative activity of sage (Salvia fruticosa mill.) under the influence of genotype and harvesting period. Ind. Crops Prod. 94, 240–250. doi: 10.1016/j.indcrop.2016.08.022

[B64] Sawchik J. Mallarino A. P. (2008). Variability of soil properties, early phosphorus and potassium uptake, and incidence of pests and weeds in relation to soybean grain yield. Agron. J. 100, 1450–1462. doi: 10.2134/agronj2007.0303

[B65] Sheng Y. He P. Xu X. Liu Y. (2023). A large-scale assessment on spatial variability of potato yield and soil chemical properties in northern China. Soil Tillage Res. 231, 105743. doi: 10.1016/j.still.2023.105743

[B66] Singh S. P. Mahapatra B. S. Pramanick B. Yadav V. R. (2021). Effect of irrigation levels, planting methods and mulching on nutrient uptake, yield, quality, water and fertilizer productivity of field mustard (Brassica rapa l.) under sandy loam soil. Agric. Water Manage. 244, 106539. doi: 10.1016/j.agwat.2020.106539

[B67] Singh S. Sakshi, Annapurna K. Shrivastava N. Varma A. (2022). Symbiotic interplay of piriformospora indica and azotobacter chroococcum augments crop productivity and biofortification of zinc and iron. Microbiol. Res. 262, 127075. doi: 10.1016/j.micres.2022.127075 35688099

[B68] Sintim H. Y. Shahzad K. Bary A. I. Collins D. P. Myhre E. A. Flury M. (2022). Differential gas exchange and soil microclimate dynamics under biodegradable plastic, polyethylene, and paper mulches. Ital. J. Agron. 17. doi: 10.4081/ija.2022.1979

[B69] Smets T. Poesen J. Bochet E.. (2008). Impact of plot length on the effectiveness of different soil-surface covers in reducing runoff and soil loss by water. Prog. Phys. Geography: Earth Environ. 32, 654–677. doi: 10.1177/0309133308101473

[B70] Somanathan H. Sathasivam R. Sivaram S. Mariappan Kumaresan S. Muthuraman M. S. Park S. U. (2022). An update on polyethylene and biodegradable plastic mulch films and their impact on the environment. Chemosphere 307, 135839. doi: 10.1016/j.chemosphere.2022.135839 35961455

[B71] Thankamani C. K. Kandiannan K. Hamza S. Saji K. V. (2016). Effect of mulches on weed suppression and yield of ginger (Zingiber officinale roscoe). Scientia Hortic. 207, 125–130. doi: 10.1016/j.scienta.2016.05.010

[B72] Thakur M. Kumar R. (2021). Mulching: Boosting crop productivity and improving soil environment in herbal plants. J. Appl. Res. Med. Aromat. Plants 20, 100287. doi: 10.1016/j.jarmap.2020.100287

[B73] Tsuboi S. Yamamoto-Tamura K. Takada A. Yonemura S. Takada Hoshino Y. Kitamoto H.. (2022). Selection of p-nitrophenyl fatty acid substrate suitable for detecting changes in soil esterase activity associated with degradation of biodegradable polyester mulch films: A field trial Authors Shu. Ital J. Agron. 17, 2040. doi: 10.4081/ija.2022.2040

[B74] Varol M. (2020). Spatio-temporal changes in surface water quality and sediment phosphorus content of a large reservoir in Turkey. Environ. pollut. 259, 113860. doi: 10.1016/j.envpol.2019.113860 31887594

[B75] Vijayaraghavareddy P. Xinyou Y. Struik P. C. Makarla U. Sreeman S. (2020). Responses of lowland, upland and aerobic rice genotypes to water limitation during different phases. Rice Sci. 27, 345–354. doi: 10.1016/j.rsci.2020.05.009

[B76] Wang F. X. Wu X. X. Shock C. C. Chu L. Y. Gu X. X. Xue X. (2011). Effects of drip irrigation regimes on potato tuber yield and quality under plastic mulch in arid Northwestern China. Field Crops Res. 122, 78–84. doi: 10.1016/j.fcr.2011.02.009

[B77] Wen Y. Wu X. Liu J. Zhang J. Song L. Zhu Y.. (2023). Effects of drip irrigation timing and water temperature on soil conditions, cotton phenological period, and fiber quality under plastic film mulching. Agric. Water Manage. 287, 108435. doi: 10.1016/j.agwat.2023.108435

[B78] Wilkinson L. (2011). ggplot2: Elegant graphics for data analysis by WICKHAM, H. Biometrics. 67, 678–679. doi: 10.1111j.1541-0420.2011.01616.x

[B79] Wszelaczyńska E. Pobereżny J. Dudek S. Kuśmierek-Tomaszewska R. Żarski J. Pawelzik E. (2015). The effects of fertilizers, irrigation and storage on the properties of potato tubers and their constituent starches. Starch - Stärke 67, 478–492. doi: 10.1002/star.201400196

[B80] Xing Y. Zhang T. Jiang W. Li P. Shi P. Xu G.. (2022). Effects of irrigation and fertilization on different potato varieties growth, yield and resources use efficiency in the Northwest China. Agric. Water Manag. 261, 107351. doi: 10.1016/j.agwat.2021.107351

[B81] Xiong L. Wu W. (2022). Can additional agricultural resource inputs improve maize yield, resource use efficiencies and emergy based system efficiency under ridge-furrow with plastic film mulching? J. Cleaner Production 379, 134711. doi: 10.1016/j.jclepro.2022.134711

[B82] Yang W. Chang F. Ma D. Wang S. Yin L. (2022). Subsoil water use to attain stable high yields of winter wheat in drylands Loess Plateau of China. Eur. J. Agron. 139, 126558. doi: 10.1016/j.eja.2022.126558

[B83] Yavuz D. Seymen M. Yavuz N. Çoklar H. Ercan M. (2021). Effects of water stress applied at various phenological stages on yield, quality, and water use efficiency of melon. Agric. Water Manage. 246, 106673. doi: 10.1016/j.agwat.2020.106673

[B84] Zhang R. Lei T. Wang Y. Xu J. Zhang P. Han Y.. (2022a). Responses of yield and water use efficiency to the interaction between water supply and plastic film mulch in winter wheat-summer fallow system. Agric. Water Manage. 266, 107545. doi: 10.1016/j.agwat.2022.107545

[B85] Zhang S. Li P. Wei Z. Cheng Y. Liu J. Yang Y.. (2022b). Cyperus (Cyperus esculentus L.): A review of its compositions, medical efficacy, antibacterial activity and allelopathic potentials. Plants 11, 1127. doi: 10.3390/plants11091127 35567128 PMC9102041

